# Acquired perforating dermatosis in a patient with chronic renal failure*

**DOI:** 10.1590/abd1806-4841.20164619

**Published:** 2016

**Authors:** Karen de Almeida Pinto Fernandes, Lourenço de Azevedo Lima, Juliana Chaves Ruiz Guedes, Ricardo Barbosa Lima, Antônio Macedo D'Acri, Carlos José Martins

**Affiliations:** 1 Universidade Federal do Estado do Rio de Janeiro (UNIRIO) - Rio de Janeiro (RJ), Brazil; 2 Private clinic - São Paulo (SP), Brazil

**Keywords:** Collagen, Dermis, Elastic tissue, Epidermis, Renal dialysis, Renal insufficiency, chronic

## Abstract

Perforating dermatoses are a group of skin diseases characterized by transepidermal elimination of dermal material. The disease is divided into two groups: the primary group and the secondary group. The classical or primary perforating dermatoses are subdivided into four types according to the eliminated dermal materials: Kyrle disease, perforating reactive collagenosis, elastosis perforans serpiginosa, and perforating folliculitis. The secondary form is known as acquired perforating dermatosis. The term was proposed in 1989 by Rapini to designate the perforating dermatoses affecting adult patients with systemic disease, regardless of the dermal materials eliminated. This report describes a case of the disease with elimination of collagen and elastic fibers in a patient with chronic renal failure.

## INTRODUCTION

Perforating dermatoses are a group of disorders characterized by transepidermal elimination of dermal material.^1^ They are divided into primary (or classic) and secondary forms.^1^ Primary perforating dermatoses are subdivided into Kyrle disease (KD), reactive perforating collagenosis (RPC), perforating serpiginous elastosis (PSE), and perforating folliculitis (PF), according to the eliminated dermal material.^2^ The elimination of cellular debris without collagen or elastic fibers is seen in KD; RPC eliminates only collagen fibers; EPS eliminates only elastic fibers; PF eliminates damaged hair follicles.^2^

The secondary form of the disease is known as acquired perforating dermatosis (APD).^1^ The term was proposed by Rapini in 1989 to designate the perforating dermatosis affecting adult patients with chronic renal failure (CRF), diabetes mellitus (DM), and, more rarely, other systemic diseases, regardless of the eliminated dermal material.^2^ APD is a little-known disease with a still controversial etiology.^3^ Its pathophysiology is uncertain and it is believed that several factors participate in the process.^4^ Clinical and histological features are not uniform and may resemble any of the four perforating disorders, in isolation or as a combination of them.^5^ We report a case of APD with elimination of collagen and elastic fibers in patients with CRF.

## CASE REPORT

**Figure 1:**
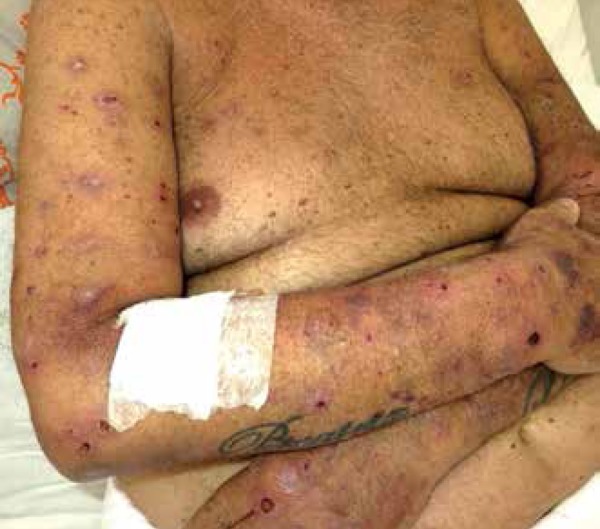
Panoramic view showing papules and erythematous nodules with central keratotic plugs and scarring lesions on the trunk and upper limbs

**Figure 2:**
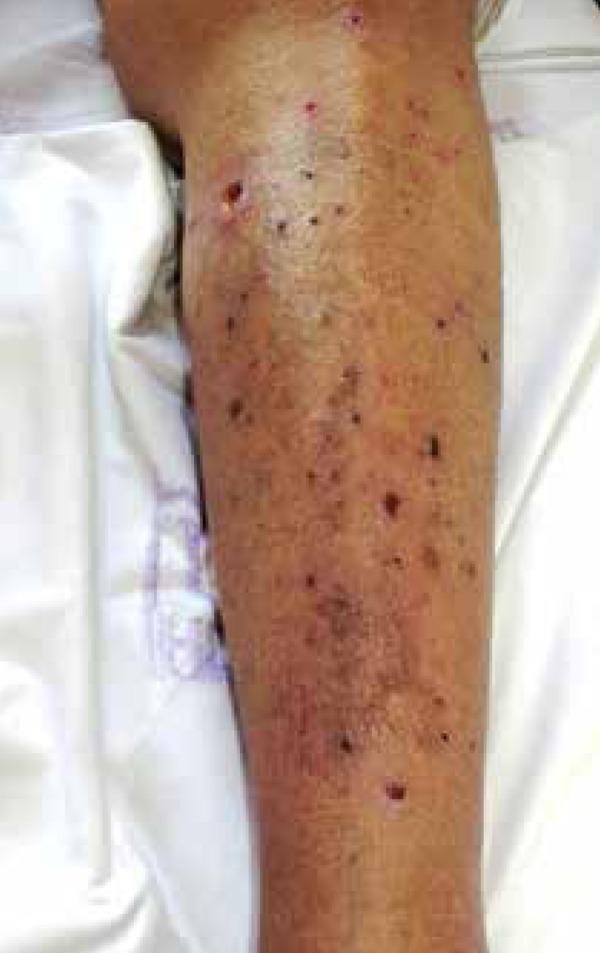
On the right leg, erythematous papules and nodules with volcano- like center and central keratotic plug. Some injuries reveal signs of secondary infection

**Figure 3:**
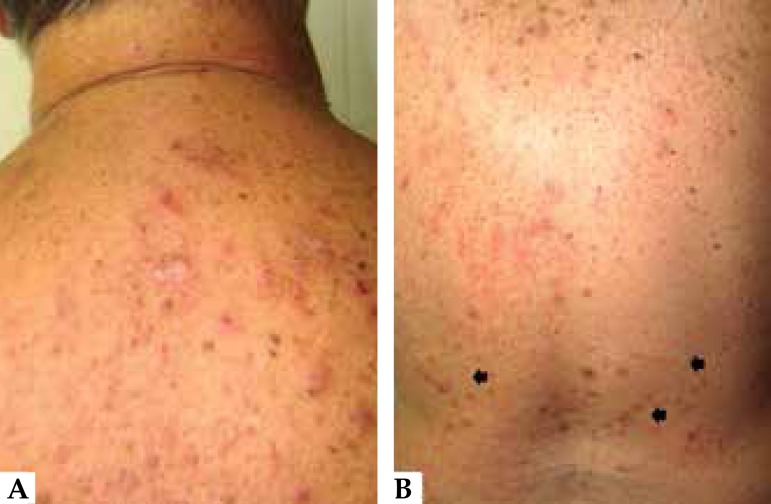
**A)** In the dorsal region, erythematous papules and excoriations, some covered with crusts. **B)** In the lumbar region, erythematous papules, some following a linear path, suggesting Koebner phenomenon (arrows)

**Figure 4:**
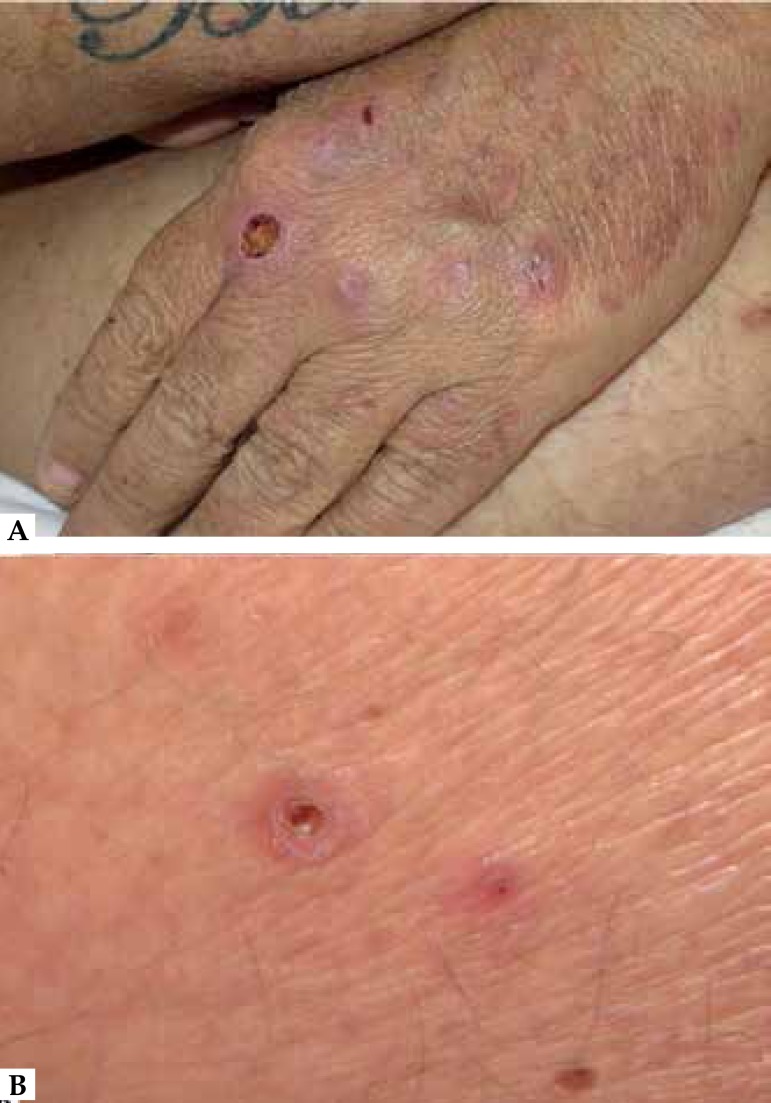
In more detail, lesions with peculiar feature **(A)** on the dorsum of the left hand and **(B)** posterior part of the left knee

**Figure 5:**
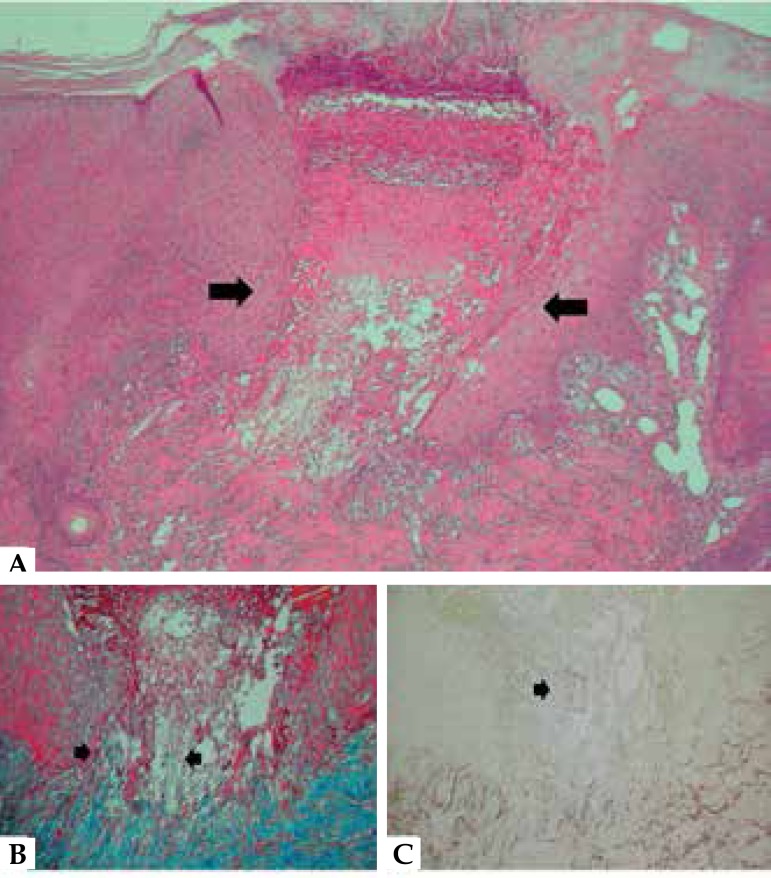
**A)** Invagination of the epidermis filled with plugs composed of keratin and serofibrinous exudate, and rupture area with epithelial hyperplasia at the edges, where the dermal material is eliminated (HE 100x) **B)** Masson’s trichrome stain showing the elimination of collagen fibers in blue (100x). **C)** Orcein stain showing the elimination of elastic fibers in brown (100x)

We report a 57-year-old male patient who presented with generalized pruritus for about six months, resulting in skin lesions on the upper limbs, lower limbs, and trunk. Pathological history revealed systemic arterial hypertension and CRF, and the patient was on hemodialysis for three years. Dermatological examination revealed erythematous papules and nodules with a keratotic center on the upper limbs, lower limbs, and trunk, some with signs of secondary infection (Figures 1-3). In the lumbar region, we observed erythematous papules, some in linear arrangement suggesting Koebner phenomenon (Figure 3). Umbilicated papular lesions, with central keratotic plugs, as seen on the back of the left hand and posterior part of the left knee, had the same predominant morphological features of the dermatological picture (Figure 4). Histopathological examination revealed ruptured epidermis with elimination of dermal material (Figure 5). Masson's trichrome and orcein stains showed elimination of degenerated collagen and elastic fibers (Figure 5). Laboratory tests highlighted the sharp rise in urea and serum creatinine. Initially, the patient was submitted to outpatient treatment with antihistamines and oral antibiotics, with partial improvement. However, the lesions worsened afterwards, revealing severe infection. The patient was treated with intravenous antibiotics and allopurinol 100mg daily, showing partial improvement, but evolved to death due to cardiovascular complications.

## DISCUSSION

For over two decades, the four largest perforating dermatoses were observed in adult patients with CRF and DM.^2^ Authors used different confusing nomenclatures, such as acquired reactive perforating collagenosis, reactive perforating collagenosis in DM and in renal failure, perforating folliculitis in patients on *hemodialysis*, uremic follicular hyperkeratosis, among others.^2^ Due to the clinical and pathological similarity of the cases, it was suggested that they would be variants of the same process.^2^ In 1989, Rapini et al. reported the cases of four patients reveling the elimination of both collagen and elastic fibers.^6^ This finding led them to suggest the term APD for all perforating dermatoses that affect adult patients with chronic kidney disease, diabetes, and other systemic diseases.^6^ Pathophysiology is still uncertain, but it is believed that chronic pruritus in predisposed patients may cause the rupture of collagen fibers with consequent elimitation.^2,4,5^ Diabetic microangiopathy may contribute to collagen damage and to the microdeposition of substances that are not removed by dialysis, causing local inflammatory reactions.^2,4,5^ In addition, leukocyte infiltration seen in lesions secretes interleukin-1, which stimulates the synthesis of metalloproteinases, degrading the components of the extracellular matrix.^5^

Even after the proposal of the term APD by Rapini, we found differences in the nomenclature used in the literature, with reports designating the acquired form based on the type of eliminated dermal material, making comprehension and bibliographic research more difficult. In our case, we observed elimination of collagen and elastic fibers as in the four cases described by Rapini.^6^ In a study of 22 APD cases, Saray et al. found histological features similar to KD in most patients (45.5%), followed by RPC (36.4%), perforating folliculitis (13.63%), and PSE (4.54%). 72% of patients with APD have chronic kidney disease.^7^ On average, 11% of patients with APD are on dialysis.^8^ APD predominates in males, and the usual age of onset of lesions is at 56 years.^7^ The main symptom is pruritus, and Koebner phenomenon is frequent in most cases.^7^ The most common complication is the secondary infection of the lesions, as occurred in our patient.^7^ Differential diagnoses include: nodular prurigo; excoriated dermal diseases (such as granuloma annulare, and lichen planus); perforating pseudoxanthoma elasticum; hyperkeratosis lenticularis perstans (Flegel's disease), and other classical perforating dermatoses.^9^ Diagnosis is based on the patient's history, clinical appearance of the lesions, and histopathology. Among the many treatment options suggested for perforating dermatoses, recent studies have reported good results with allopurinol since this drug inhibits the action of the enzyme xanthine oxidase and thus reduces the synthesis of free radicals that damage collagen.^5,10^ Other treatment options described in the literature include: phototherapy (UVB-NB); topical, oral, and intralesional corticosteroids; antihistamines; topical and oral retinoids; methotrexate; and doxycycline.^9^
